# Is there a relationship between sleep apnoea and microalbuminuria?

**DOI:** 10.1007/s11325-021-02461-6

**Published:** 2021-08-13

**Authors:** Melek Cihanbeylerden, Melike Bağnu Yüceege

**Affiliations:** 1 Ağrı Research and Training Hospital, Ağrı, Turkey; 2 Faculty of Health Sciences, Diskapi Yildirim Beyazit Research and Training Hospital, Ankara, Turkey

**Keywords:** Obstructive sleep apnoea, Microalbuminuria, Endothelial damage, Polysomnography

## Abstract

**Introduction:**

Obstructive sleep apnoea (OSA) is a cause of hypoxia, and the correlation between hypoxia and microvascular complications is well known. Microalbuminuria (MAU) is a marker for endovascular dysfunction and an indicator of cardiovascular events and all-cause mortality in the general population. The aim of this study was to investigate the relationship between microvascular damage and the metabolic complications of OSA based on the presence of MAU.

**Material and method:**

Urinary albumin/creatinine ratio (ACR) and microalbumin level were examined in patients with an apnoea-hypopnoea index (AHI) greater than 5/h (study group) and in patients with an AHI less than 5/h (control group). The exclusion criteria were other possible causes of MAU (hypertension, nephropathy, coronary artery disease, and severe thyroid dysfunction).

**Results:**

Of 103 patients enrolled, 80 formed the group with OSA and 23 served as controls. According to the AHI values, the patients were divided into four groups as normal, mild, moderate and severe. There was no significant difference between the four groups in terms of the microalbumin level and urinary albumin/creatinine ratio.

**Conclusion:**

In this study, no significant relationship was found between MAU and sleep apnoea.

## Introduction and aim

The most important complications of patients with obstructive sleep apnoea (OSA) are related to the cardiovascular system, and the cause of the underlying pathophysiology of this relationship is not yet fully known. In recent years, OSA and cardiovascular disease (CVD) have been observed to have a relationship with pathophysiological mechanisms, such as oxidative stress, sympathetic activation, endothelial dysfunction and inflammation. There are many studies suggesting that hypoxia seen in OSA may lead to metabolic events through increased inflammation in the respiratory system and systemic circulation [1].

Daily urinary albumin excretion is below 30 mg under physiological conditions. The presence of 30–300 mg of albumin in a 24-h urine sample is defined as microalbuminuria (MAU) [2]. MAU is considered to be associated with endothelial dysfunction, and there are studies showing that it may be a marker of renal endothelial damage that can be detected in the early period of CVD [3]. It is known that the risk of CVD increases in OSA [4]. There are similar pathophysiological mechanisms observed in OSA and involved in the formation of MAU, and some previous studies have reported a relationship between OSA and MAU; however, it remains unknown to what extent this relationship is associated with the complication of OSA, including hypertension (HT), diabetes mellitus (DM) and coronary artery disease (CAD) [5]. In addition, other studies evaluating the relationship between MAU and OSA have presented conflicting results [6]. MAU has been used in the regulation of treatment in patients with DM and has reduced the risk of CVD and renal failure in this population [7]. Albumin measurement in urine, as an inexpensive, safe and readily available method, can play a role in the complication management in OSA, similar to its role in the management of DM.

Studies have also shown that Continuous Positive Airway Pressure (CPAP) therapy improves systemic inflammation in OSA [8]. In our study, we aimed to examine the relationship between MAU and intermittent hypoxia that occurs in patients with OSA.

## Material and method

This study was conducted in the Chest Diseases and Sleep Disorders Centre of Health Sciences University Diskapi Yildirim Beyazit Health Application and Research Centre between June 2019 and September 2019. Ethical approval was obtained from the Planning and Coordination Board of the university with the decision number 62/29.

### Patient selection

This study involved the prospective evaluation of a total of 103 patients with the complaints of excessive daytime sleepiness, fatigue, snoring, respiratory arrest at night, breath holding and feeling of choking when waking.

A detailed history was taken from the patients with suspected OSA, and a physical examination was performed. Additional diseases were questioned, and the Epworth Sleepiness Questionnaire was administered. The pulmonary function test was applied, and routine blood tests were requested. The patients were informed about the study and signed consent forms confirming that they agreed to participate in the study. Spot urine samples were requested from the volunteer patients on the day they were scheduled to undergo polysomnography (PSG). In both patient groups with and without OSA, the albumin-to-creatine ratio (ACR) in spot urine and the urinary microalbumin level were evaluated.

For all the patients in the OSA and control groups, identity information, demographic characteristics (age, gender, height, weight and body mass index), anthropometric characteristics (waist-hip-neck circumference, known diseases, smoking history, pulmonary function test results, PSG results and ACR and microalbumin level in spot urine as well as 24-h urinary microalbumin values in selected patients were recorded.

### Exclusion criteria

Patients with a previous diagnosis of HT, DM, CAD, congestive heart failure, acute-chronic renal failure or nephropathies, chronic obstructive pulmonary disease (COPD) or asthma, those with known overt thyroid dysfunction, those with a fasting blood glucose of 126 mg/dl or more on the day of the PSG appointment, patients receiving treatment for OSA and those that did not agree to participate in the study were excluded from the sample.

### Polysomnography

Before attending the PSG appointment, the patients were asked to take a shower, to shave their beard if male, to stop the use of any drugs that could affect their sleep if any and to stop taking hypnotic drugs, if any, at least 5 to 10 days earlier. They were also asked not to drink alcohol and caffeinated drinks on the day of the study and not to sleep during the day if possible. The PSG examination was performed with the Compumedics (44-channel E-series, Australia) device with patients staying at the Sleep Disorder Centre of the hospital overnight. During the PSG examination, electroencephalography (C3-A2, C4-A1, O1-A2 and F4-A1), two-channel electrooculography and electromyography recording (from the jaw and tibialis anterior muscle), oronasal air flow, thoracic and abdominal movements and body position were recorded, and oxygen saturation was measured from the fingertip using pulse oximetry. PSG findings, including total sleep time, time to fall asleep, sleep stages (stage 1, stage 2, stage 3 and REM), oxygen desaturation index (ODI), minimal oxygen saturation (MOS), apnoea hypopnoea index (AHI), respiratory disturbance index (RDI), apnoea index, hypopnoea index and arousal index were recorded. The PSG data were scored by a pulmonologist according to the American Sleep Disorders Association criteria. The AHI value was calculated using the number of apnoeas and hypopnoeas. Patients with an AHI of < 5 were considered to have normal findings, while those with an AHI of 5–15 were evaluated as having mild OSA, 16–30 moderate OSA and > 30 severe OSA.

### Biochemical analyses

For urine creatinine and albumin analyses, spot urine was used as a screening test since the collection and standardisation of 24-h urine samples were difficult. However, the measurement of albumin in spot urine alone may cause false results due to the dilution or concentration of urine. In order to prevent these errors, ACR was calculated, and the microalbumin level was evaluated according to this ratio. The reference ranges were accepted as 30–300 mg/day for ACR in spot urine, 30–300 mg/day for MAU and 30–300 mg/24 h for the MAU level in 24-h urine. Racial and gender-related factors were not included in the evaluation.

The urinary microalbumin level was measured with the turbidimetric method using the AU 5800 Beckman Coulter autoanalyzer and the Beckman Coulter urine/albumin kit. The creatinine level was measured with uncompensated kinetic Jaffe method using the same autoanalyzer and the Beckman Coulter creatinine kit.

### Statistical analysis

IBM Statistical Package for the Social Sciences (SPSS) v. 20 was used for statistical analyses. The Kolmogorov–Smirnov test was used to check the conformance of the data to normal distribution. Continuous data conforming to normal distribution were given as mean ± standard deviation, and those not conforming to normal distribution were expressed as median and minimum–maximum values, while categorical data were obtained as percentages (%). The Mann–Whitney *U* test was conducted to compare the data of two groups for data that were not normally distributed, and the independent-samples *t* test was undertaken for the comparison of normally distributed data between two groups. The Kruskal–Wallis test was used to compare the non-parametric data of more than two independent groups. The *χ*^2^ or Fisher’s exact test was utilised to compare independent categorical variables. Pearson’s and Spearman’s correlation analyses were performed for the correlation analysis of continuous data, and *p* < 0.05 was considered statistically significant.

## Results

The study included 103 patients, aged 15 to 69 years, 76 (74%) men. In each of the two groups, the number of men was higher than that of women. The proportion of men was higher in the OSA group compared to the control group (*p* < 0.001). The mean body mass index, neck circumference and waist circumference values were higher in the OSA group compared to the control group (*p* = 0.001, *p* < 0.001, and *p* < 0.001, respectively). There were a total of 45 (43%) active smokers. Smoking histories were similar in the two groups. The comparison of the demographic characteristics of the OSA and control groups is shown in Table 1.

**Table 1:** Comparison of the demographic characteristics of the OSA and control groups

	OSA (AHI ≥ 5) *n* = 80	Control (AHI < 5) *n* = 23	
Age, years, mean ± SD	45.0 ± 10.5	42.0 ± 10.0	0.217
Gender			
Male, *n* (%)	68 (85)	8 (35)	< 0.001
Female, *n* (%)	12 (15)	15 (65)	
BMI, kg/m^2^, mean ± SD	31.0 ± 5.2	27.0 ± 5.7	0.001
Neck circumference, cm, mean ± SD	41 ± 3.7	36 ± 4.1	< 0.001
Waist circumference, cm, mean ± SD	105 ± 12.4	93 ± 11.9	< 0.001
Smoking			
Non-smoker, *n* (%)	26 (33)	7 (30)	0.897
Active smoker, *n* (%)	34 (42)	11 (48)	
Ex-smoker, *n* (%)	20 (25)	5 (22)	

*OSA* obstructive sleep apnoea, *AHI* apnoea-hypopnoea index, *BMI* body mass index

**Figure1:**
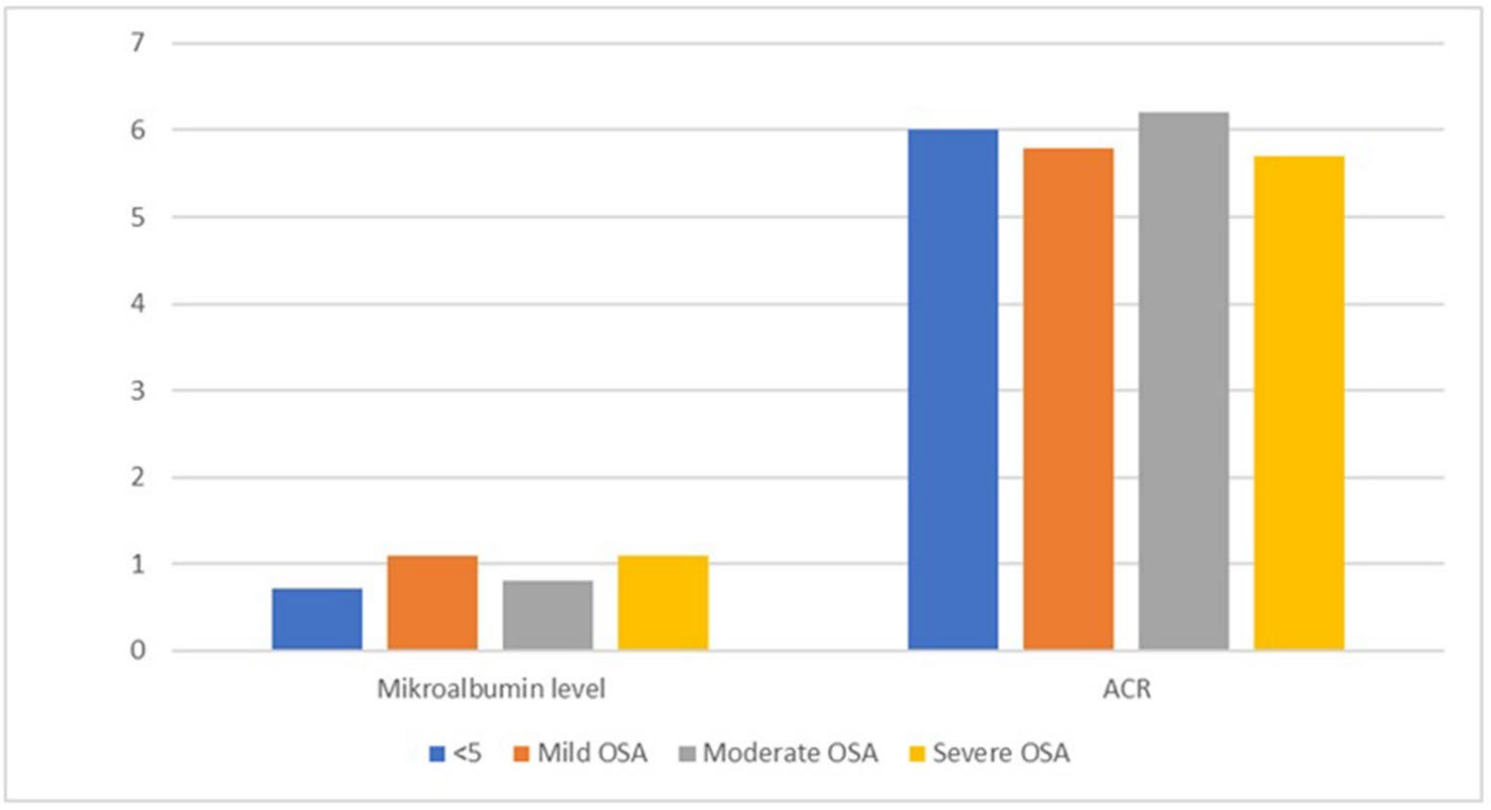
Microalbumin level and albumin/creatinine ratio (ACR) according to the apnoea-hypopnoea index

The patients with an AHI index of < 5 were considered to have normal findings while an AHI = value of 5–15 was evaluated as mild OSA, 16–30 as moderate OSA and > 30 as severe OSA. There were 28 patients in the mild OSA group, 26 in the moderate OSA group and 26 patients in the severe OSA group. When the patients were grouped according to AHI, there was no significant difference between the four groups in terms of the microalbumin level and ACR (*p* = 0.618 and *p* = 0.890, respectively). The paired comparison of these groups revealed that the microalbumin level of the AHI < 5 group did not significantly differ from the OSA group (*p* = 0.785) or the severe OSA group (*p* = 0.631). There was no significant difference between the moderate and severe OSA groups and the AHI < 5 group in terms of the microalbumin level and ACR (*p* = 0.663 and 0.617, respectively). The comparison of the microalbumin level and ACR of the AHI groups is shown in Table 2 and Fig. 1.

**Table 2:** Comparison of the microalbumin level and ACR between the AHI groups

	AHI < 5 normal	5 ≤ AHI ≤ 15 mild OSA	16 ≤ AHI ≤ 30 moderate OSA	AHI > 30 severe OSA	*p* value
Microalbumin level (mg/day), median (IQR)	0.71 (0.36–1.40)	1.10 (0.84–1.37)	0.81 (0.62–1.33)	1.10 (0.51–1.57)	0.340
ACR (mg/day) median (IQR)	6.0 (3.9–10.9)	5.8 (4.3–7.7)	6.2 (4.6–7.9)	5.7 (4.6–10.6)	0.974

*ACR* albumin/creatinine ratio, *AHI* apnoea-hypopnoea, *IQR* interquartile range

There was a low-level linear correlation between ACR and age. However, no correlation was detected between ACR and the remaining parameters, namely, body mass index, AHI, percentage of oxygen desaturation, awake saturation level, 3% ODI, 4% ODI, obstructive apnoea, mixed apnoea, central apnoea, number of hypopnoeas and total number of apnoeas. The results of the correlation analyses between ACR and other parameters are shown in Table 3.

**Table 3:** Correlation analysis between ACR and other parameters

	ACR (mg/day)	
	*R*	*p*
Age	0.272	0.005
BMI	− 0.012	0.903
AHI	− 0.008	0.934
Oxygen desaturation percentage	− 0.077	0.441
Awake oxygen saturation	− 0.041	0.682
ODI 3%	− 0.011	0.915
ODI 4%	− 0.020	0.844
Apnoea index	− 0.155	0.119
Hypopnoea index	0.031	0.757
Obstructive apnoea	− 0.122	0.221
Mixed apnoea	− 0.128	0.199
Central apnoea	− 0.131	0.188
Number of hypopnoeas	0.012	0.904
Total number of apnoeas	− 0.162	0.102

*ACR* albumin/creatinine ratio, *BMI* body mass index, *AHI* apnoea-hypopnoea index, *ODI 3%* 3% oxygen desaturation index, *ODI 4%* 4% oxygen desaturation index

## Discussion and conclusion

In our study, we investigated whether OSA was an independent risk factor for MAU by excluding patients with risk factors that could cause MAU (except for obesity). There was no significant difference in the microalbumin level and ACR between the control group and the patients admitted to our Sleep Disorder Centre with OSA complaints and diagnosed with OSA based on PSG. The patients were grouped according to AHI. There was no significant difference between these four groups in terms of the microalbumin level and ACR. A low-level linear correlation was observed between ACR and age. There was also a very low level of inverse correlation between ACR and the total apnoea count. No significant relationship was observed between he urinary microalbumin level and apnoea count.

In a study conducted with 91 morbidly obese adults who underwent routine PSG before bariatric surgery, it was reported that MAU was significantly associated with diastolic blood pressure, and the presence or severity of OSA had no effect on albuminuria [9]. Another study included 75 patients and examined day and night proteinuria. Patients with renal failure, DM or systemic lupus erythematosus and those using angiotensin-converting enzyme inhibitors and angiotensin receptor blockers were excluded. The authors stated that there was no significant relationship between the severity of sleep apnoea and the urine microalbumin values measured during both sleep and awake periods [6].

Some studies have found a relationship between OSA and MAU. However, the results are controversial and confusing since some of these studies included patients possessing risk factors that may cause overt MAU, such as HT and DM [5, 10]. In a study by Faulx et al., the glomerular filtration rate and MAU values of the patients were examined, and it was reported that there was an increase in albumin excretion in patients with severe OSA, which was interpreted as a marker of endothelial dysfunction in cardiovascular events. The authors described a limitation of their study as the presence of HT and obesity in the group with severe OSA [10]. In that study, which found a relationship between OSA and MAU, HT was not excluded from comorbidities that could cause MAU. In a different study, the relationship between OSA and MAU was examined in 507 elderly male patients, and a significant relationship was observed between RDI and ACR and between nocturnal hypoxia and ACR, but this was considered to be associated with BMI and presence of DM and HT in patients [11]. Therefore, that study was also limited due to the inclusion of patients with HT and DM since this could mask the real relationship between OSA and MAU. When evaluated carefully, the common aspect of studies finding a relationship between MAU and OSA can be summarized as not excluding patients with comorbidities.

Undiagnosed OSA has been reported in most patients with idiopathic HT [12]; therefore, HT can be considered a common complication of OSA. While MAU is a common marker in HT, it is confusing that hypertensive patients were not excluded in studies examining the relationship between OSA and MAU. In our study, we excluded patients with HT in order not to lead to confusion concerning two different concepts of MAU: one as an indicator of hypertensive nephropathy and the other as an indicator of nephropathy caused by OSA.

There are also studies conducted with patients with OSA, in which the patient group that may be at risk of MAU was excluded from the sample, and a significant relationship was found between OSA and MAU [13, 14]. In a study conducted in Taiwan with 40 OSA cases, patients with HT, DM, abnormal kidney function, liver cirrhosis, COPD, haematological disease, autoimmune disease, cancer or recent infection were excluded from the study. The authors reported a significant relationship between ACR and OSA severity and duration of oxygen desaturation during sleep. However, that study did not perform a MAU evaluation in 24-h urine samples in patients with a significantly higher ACR according to the analysis of morning urine [13]. In our study, two patients in the AHI ≥ 5 group had an ACR of > 30 mg/day, and as a result of 24-h urine sampling of these two patients, we found that their microalbumin levels were normal. In another study, patients with a history of DM, HT, kidney failure, heart failure, CAD, collagen tissue disease, high serum creatinine and urine infection and those using angiotensin-converting enzyme inhibitors were excluded from the analysis. In the evaluation performed on 35 patients and 11 control groups, it was reported that MAU was associated with the length of time in oxygen desaturation. Low-grade ACR (0–30 mg/mmol) was found to be associated with OSA in normotensive patients without DM, regardless of age and BMI [10]. However, the number of patients was small and the urine MAU measurement was evaluated based on only a single urine sample, and a 24-h urine sample was not studied. In our study, spot urine samples were examined once, but the microalbumin level was measured in 24-h urine sampling in two patients with an ACR of > 30 mg/day.

Due to the frequent night-time urination in patients with OSA and DM, analysing MAU in the first morning urine raises concerns about the standardisation of the samples. In our study, morning blood and urine samples were requested for the patients on the day of their PSG appointment. Our urine collection method was not in the form of the first morning urine sample. We investigated ACR and the microalbumin level in the spot urine sample given at any hour in the morning. In addition, 24-h urine MAU sampling was requested for the two patients whose spot urinary ACR and microalbumin levels were found to be suspicious. Due to the conflicting results in the literature, clinicians should request an MAU measurement in 24-h urine samples for the investigation of nephropathies in patients with suspicious spot urine results [15]. The American Diabetes Association states that clinicians should detect the presence of albuminuria in two or three different urine samples taken within 3 to 6 months to make a diagnosis of this condition. Urinary albumin excretion is affected in cases with marked hyperglycaemia, heart failure, urinary tract infection, haematuria and uncontrolled HT; those that have undertaken high-intensity exercises within the last 24 h; and those with acute febrile illness [16].

There is no definite consensus on the urinary ACR value in the definition of spot urine MAU. The 2012 Clinical Practice Guideline for the Evaluation and Management of Chronic Kidney Disease reported the ACR value for MAU as 30–300 mg/day, which is also the range on which we based our evaluation. However, in the literature, different normal values were calculated for MAU as influenced by gender and racial factors, ACR was measured in urine only once, and MAU was not measured in 24-h urine [5]. If ACR evaluated based on the single-time analysis of spot urine or urine collected at a specific time provided significant results, the lack of the confirmation of this findings using 24-h urine sampling may lead to an increase in the number of patients in the case group. While defining MAU in our study, ACR was not affected by racial and gender-related factors. Considering the possibility of false positive ACR measurement in spot urine due to the presence of certain conditions, we also measured the microalbumin level in 24-h urine samples in two cases with an AHI of ≥ 5. We found their microalbumin levels to be normal. We also determined that the 24-h urine and spot urine microalbumin levels of both cases were inconsistent.

The strength of our study is that all patients with comorbidities that could cause MAU were excluded from the study. Furthermore, PSG, which is the gold standard method in the diagnosis of OSA, was applied to all patients participating in the study. Lastly, 24-h urine sampling was also undertaken in patients detected to have MAU in spot urine. The limitation of our study can be considered the small number of patients with severe OSA.

In conclusion, clinically different results may be obtained in patients with prolonged exposure to intermittent hypoxia or in those with severe OSA. Prospective studies involving more patients may further clarify the relationship between MAU and OSA.

## References

[1] Resuli AS (2019) Cardiovascular Complications in obstructive sleep apnea syndrome. Multidiscip Cardiovasc Ann 10(1)

[2] Jerums G, Maclsaac RJ (2002). Treatment of microalbuminuria in patients with type 2 diabetes mellitus. Treat Endocrinol.

[3] Ferris M (2007). Obesity, albuminuria, and urinalysis findings in US young adults from the Add Health Wave III study. Clin J Am Soc Nephrol.

[4] Strauss RS, Brwoner WS (2000). Risk for obstructive sleep apnea. Ann Intern Med.

[5] Bulcun E (2015). Microalbuminuria in obstructive sleep apnea syndrome. Sleep Breath.

[6] Mello P (2004). Night and day proteinuria in patients with sleep apnea. Am J Kidney Dis.

[7] Klausen K (2004). Very low levels of microalbuminuria are associated with increased risk of coronary heart disease and death independently of renal function, hypertension, and diabetes. Circulation.

[8] Campos-Rodriguez F (2007). Long-term effect of continuous positive airway pressure on BP in patients with hypertension and sleep apnea. Chest.

[9] Agrawal V (2009). Albuminuria and renal function in obese adults evaluated for obstructive sleep apnea. Nephron Clin Pract.

[10] Faulx MD (2007). Obstructive sleep apnea is associated with increased urinary albumin excretion. Sleep.

[11] Canales MT (2011). Sleep-disordered breathing and urinary albumin excretion in older men. Sleep Breath.

[12] Fletcher EC, Debenhke RD, Lovoi MS, Gorin AB (1985). Underdiagnosedsleep apnea in patient with essential hypertension. Ann Intern Med.

[13] Chou Y-T (2011). Obstructive sleep apnea: a stand-alone risk factor for chronic kidney disease. Nephrol Dial Transplant.

[14] Ursavas A (2008). Low-grade urinary albumin excretion in normotensive/non-diabetic obstructive sleep apnea patients. Sleep Breath.

[15] Mogensen CE (1995). Microalbuminuria and potential confounders: a review and some observations on variability of urinary albumin excretion. Diabetes Care.

[16] Basta E, Bakris G (2001). Choices and goals in the treatment of the diabetic hypertensive patient. Curr Hypertens Rep.

